# The Effect of Gelfoam Impregnated With Botulinum Toxin on Allergic Rhinitis

**Published:** 2019-07

**Authors:** Vahid Zand, Mohammadhossein Baradaranfar, Mohammadhossein Dadgarnia, Mojtaba Meybodian, Sedighe Vaziribozorg, Mohammad Mandegari, Nasrin Behniafard, Amrollah Dehghani

**Affiliations:** 1 *Department of Otolaryngology- Head and Neck Surgery* *, Otorhinolaryngology Research Center, Shahid Sadoughi University of Medical Sciences, Yazd, Iran.*; 2 *Department of Allergy and Clinical Immunology, * *Shahid Sadoughi University of Medical Sciences, Yazd, Iran.*

**Keywords:** Allergic rhinitis, Botulinum toxin, Gelfoam

## Abstract

**Introduction:**

This study evaluated the effect of gelfoam impregnated with botulinum toxin on the symptoms induced by allergic rhinitis.

**Materials and Methods:**

In total, 30 patients with allergic rhinitis who did not respond to common therapies were included in this clinical trial study. All patients were treated with intranasal gelfoam impregnated with botulinum toxin type a (40 unit in each side) placed in the middle meatus of each nostril. The main symptoms of allergic rhinitis were scored from zero to three by the patients. Symptoms recorded and compared before and two months after the treatment.

**Result:**

The mean age of patients was 31.03±6.9 years. The mean score for sneezing was 2.23 before the treatment which significantly decreased to 1.06 after the treatment (P<0.05). The mean scores of rhinorrhea, nasal congestion, and nasal itching were 2.53, 2.03, and 1.93, respectively, before the treatment which significantly decreased to 0.93, 1, and 0.8 after the treatment (P<0.05). No reported side effects was observed in this study.

**Conclusion:**

According to the results, treatment with gelfoam impregnated with botulinum toxin is an effective and safe method in patients who have not responded to common therapies for allergic rhinitis. Accordingly, it is recommended to relieve symptoms in patients with seasonal allergic rhinitis in order to maintain the effectiveness of this treatment at least 8 weeks.

## Introduction

  The prevalence of allergic rhinitis has increased in recent decades in eastern societies (1,2). It affects up to 40% of the population (3). Allergic rhinitis is a mucosal disease of the nose induced after exposure to indoor and outdoor allergens via IgE-mediated hypersensitivity reactions. The indoor and outdoor allergens include dust mites, insects, animals, fungi, and pollen. Symptoms included nasal congestion, nasal obstruction, sudden and frequent sneezing, watery rhinorrhea, and nasal itching (4). These symptoms can affect daily activities, sleep quality, and productivity of affected individuals (5-8). Although allergic rhinitis can impair the life quality of the affected individuals, symptoms can be kept under control with proper treatment (9,10).

There are several options for the treatment of increased nasal mucosal activity depending on allergic rhinitis pathogenesis and the patient’s symptoms. Various medications, including oral and topical antihistamines, decongestants, leukotriene antagonists, intranasal cromolyn, corticosteroids, and anticholinergics, are widely used in the treatment of allergic rhinitis.

Since a few of these treatment choices could improve this disease symptom, new options have been developed recently (9).

Botulinum toxin (BTX) is extracted from bacterium Clostridium botulinum purification (11). There are eight immunologically distinct serotypes of BTX designated as A, B, C1, C2, D, E, F, and G. (12). Recently, BTX type A has been used in different studies to treat patients with idiopathic and allergic rhinitis and it could effectively reduce the symptoms (13).

The BTX administration in the nasal cavity is performed by direct injection into the middle or lower meatus, or through gases or sponges impregnated with the toxin (14). In most studies, direct injection has been used for easier control of toxin dosing and the convenience of using direct injection; however, BTX injection can be associated with pain (15). 

Few studies evaluated the effectiveness of minimally invasive methods (using gases or sponges impregnated with toxin) on allergic rhinitis symptoms at different doses between 4-60 unit BTA. They found that nasal application of BTA induced decreasing in rhinorrhea and other allergic rhinitis symptoms and it was safer than the injection method (no side effects and pain) (11,16-18). With this background in mind, the effect of gelfoam impregnated with 40 unit BTX (another minimally invasive method) was evaluated on all allergic rhinitis symptoms in this study.

## Materials and Methods

The study protocol was approved by the Ethics Committee of and the written informed consent was obtained from the participants. In total, 30 patients with persistent (19), moderate to severe allergic rhinitis who did not respond to routine treatments, such as a steroid, and fluticasone spray for 1 year and referred to ear, nose, and throat clinics were included in this clinical trial study (Table.1).

**Table 1:** Classification of allergic rhinitis according to Allergic Rhinitis and its Impact on Asthma

**Intermittent**	**Persistent**	**Mild**	**Moderate/Severe**
Symptoms lasted <4 days a week	Symptoms lasted > 4 days a week	none of the following symptoms are present:	One or more of the following symptoms are present:
		Sleep disturbance	Sleep disturbance
		Impairment of daily activities, leisure and/or sport	Impairment of daily activities, leisure and/or sport
		Impairment of school or work	Impairment of school or work

Patients with the history of allergy to Botox or local anesthetic agents, rhinoplasty surgery, anatomical disorders of the nose, persistent asthma, malignancy, tuberculosis, diabetes mellitus, neurological disorders, and other chronic systemic diseases as well as pregnant cases were excluded from the study. Moreover, the other excluded patients were those who received any current medications except standard steroid and fluticasone treatment for allergic rhinitis. All patients were subjected to complete clinical examination and skin prick test. The process of taking a history from a patient was done and data were collected using questionnaires by one special otolaryngologist. The severity rates of four main symptoms, including sneezing, rhinorrhea, nasal congestion, and nasal itching, were scored from zero (asymptomatic) to three (severe) by the patient. Moreover, the number of tissues used per day was counted by the patient and was scored based on a four-point Likert scale (0=no, 1=mild, 2=moderate, 3= moderate to severe and 4=severe) (13,20) (Table.2).

**Table 2:** Symptom scoring systems

Score	Symptom severity	Explanation	Score	Symptom severity
**Sneezing**			Itching	
**0**	None	No symptom	0	None
**1**	Mild	1-3 episodes/day	1	Mild
**2**	Moderate	4-6 episodes/day	2	Moderate
**3**	Severe	7-10 episodes/day	3	Severe
**Rhinorrhea**			Nasal congestion	
**0**	None	Not present	0	None
**1**	Mild	1-5 tissues/day	1	Mild
**2**	Moderate	6-10 tissues/day	2	Moderate
**3**	Severe	11-20 tissues/day	3	Severe

**Fig 1:**
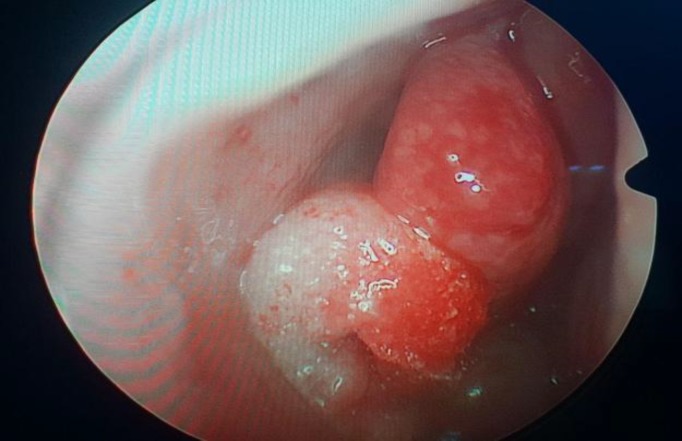
gel foam placed in each nasal cavity

All patients were treated with intranasal gelfoam (Cutanplast) impregnated with 40 unit BTX type A (Dysport® Abobotulinum toxin A, manufactured by Ipsen Biopharm Ltd. UK) in each side. The intranasal gelfoams were placed in the middle meatus of each nostril by a 0-degree rigid endoscope (Fig.1).

The gelfoam with BTX type A was absorbed and did not resolve anymore. The patients did not take any additional nasal therapy. Patients were followed up for only two months based on the duration in previous studies (8 weeks) and due to the rarity of the disease and lack of access to patients. The severity of the four main symptoms was scored by the patient and recorded by one special otolaryngologist. Adverse effects, such as nasal bleeding (epistaxis), crusting, smelling disorders, nasal dryness, and swallowing abnormalities were recorded in this study. The data were analyzed in SPSS software (Version 22).P-value less than 0.05 was considered statistically significant.

## Results

Totally, 30 patients with persistent, moderate to severe allergic rhinitis who did not respond to routine treatments were involved in this clinical trial study. The mean age of patients was 31.03±6.9 years with the age range of 18 to 45 years. Moreover, 18 (60%) patients were male. The mean score of sneezing was obtained at 2.23 before the treatment which significantly decreased to 1.06 after the treatment (P<0.05). The mean scores of rhinorrhea, nasal congestion, and nasal itching were 2.53, 2.03, and 1.93, respectively, before the treatment which significantly decreased to 0.93, 1, and 0.8 after the treatment (P<0.05) (Table.3). Table 4 presents the frequency of four main symptoms before and after the treatment.

According to the results, the frequency of sneezing was significantly decreased after the treatment. Furthermore, there were no significant differences before and after the treatment in terms of the frequency of rhinorrhea, nasal congestion, and nasal itching. However, the number of patients with severe rhinorrhea, severe nasal congestion, and severe nasal itching decreased from 20 to 1, 11 to 1 and 10 to 0, respectively, after the treatment. No side effects were reported after the treatment.

**Table 3:** Severity of allergic rhinitis before and after the treatment with gelfoam

	Before the treatment N=30	After the treatment N=30	P-value
**Sneezing**	2.23±0.89	1.06±0.82	<0.001
**Rhinorrhea**	2.53±0.73	0.93±0.78	<0.001
**Nasal congestion**	2.03±0.92	1±0.74	<0.001
**Nasal itching**	1.93±1.01	0.8±0.76	<0.001

**Table 4:** Frequency of four main symptoms before and after the treatment

**Symptoms**	**Before the treatment**				**After the treatment**				**P-value**
	**Asymptomatic**	**Mild**	**Moderate**	**Severe**	**Asymptomatic**	**Mild**	**Moderate**	**Severe**	
Sneezing	3	0	14	13	7	16	5	2	0.02
Rhinorrhea	0	4	6	20	9	15	5	1	0.51
Nasal congestion	2	6	11	11	7	17	5	1	0.08
Nasal itching	4	4	12	10	12	12	6	0	0.08

## Discussion

The BTX is a novel pharmacological agent to reduce the symptoms of allergic rhinitis. It allows a single dose application and has long-term effects from weeks to months (21). Botulinum administration in the nasal cavity is performed using a direct injection into the middle or lower concha or through gases or sponges impregnated with the toxin. In most studies, BTX is injected directly due to easier control of toxin doses and ease of use; however, its injection can be painful (15). 

Therefore, it is essential to consider new minimally invasive methods with no side effects and pain.Given the effect of BTX on reducing the symptoms of allergic rhinitis on one hand and its painful injection on the other hand, the efficiency of gelfoam impregnated with BTX was evaluated on the symptoms induced by allergic rhinitis. All patients in this study were treated with intranasal gelfoam impregnated with 40 unit BTX type A that was placed in the middle nasal meatus. The patients were examined in terms of allergic rhinitis symptoms, including sneezing, nasal congestion, nasal itching, and rhinorrhea, before and only after a two-month intervention. The follow-up period was 8 weeks due to the rarity of the disease and lack of access to patients. According to the results, the frequency of sneezing was significantly decreased after the treatment (P<0.05). There were no significant differences before and after the treatment in terms of the frequency of rhinorrhea, nasal congestion, and nasal itching which can be due to a small number of samples. However, the number of patients with severe rhinorrhea, severe nasal congestion, and severe nasal itching decreased from 20 to 1, 11 to 1, and 10 to 0, respectively, after the treatment.

In previous studies, different doses (4-60 unit) were used for injection and topical use for the treatment of patients with allergic rhinitis (21). However, 40 unit BTX was used per nasal cavity in this study. Rohrbach et al. evaluated the effect of the local application of BTX type A in a female patient with intrinsic rhinitis. In this study, 20 unit of BTX type A (Botox^®^) was inserted into each nostril using a small sponge in close contact with the lower and middle turbinates. According to the results, nasal hypersecretion became less clearly 5 days after the treatment with no side effects (11). In a similar study, Rohrbach et al. investigated the effect of BTA type A in 20 patients with allergic rhinitis (sponge soaked with 40 unit BTA type A or saline as placebo). 

The amount of secretion clearly reduced in the case group, compared to the placebo group. Moreover, sneezing was clearly reduced in the case group and nasal congestion remained unchanged (22). Wang et al. reported the degenerations of glandular epithelium and canal epithelium in nasal mucosa after the application of Merocel sponge soaked in 10 unit BTA into the left nasal cavity of 12 guinea pigs (17). 

In several studies, the injection of different doses of BTX (20-75 unit BTX type A) into the inferior and middle turbinates of patients with persistent allergic rhinitis symptoms showed prolonged effectiveness (8-12 weeks) and efficacy for the treatment of rhinorrhea, congestion, sneezing, and itching (15,21,23-25). 

With regard to the side effects of BTX administration, Laswaki reported nasal dryness (2 cases). Moreover, Hashemi et al. pointed out to epistaxis (4%) in addition to nasal dryness (4%) (24). However, several previous studies similar to our studies revealed no nasal dryness, epistaxis, or other complications following BTX administration (11,13,23).

All of the above-mentioned studies with different follow-up periods (2-3 months) approved that injection or topical administration of BTX type A can reduce the severity of sneezing, rhinorrhea, and nasal congestion in resistant cases of allergic rhinitis. The findings of the mentioned studies are consistent with the results of this study. 

**Limitations**

Although this study paved the way for the treatment of patients with allergic rhinitis symptoms, it suffers from some limitations. The sample size of the study was small and it lacked a control group. In addition, the follow-up period was limited to 8 weeks due to lack of proper access to the patients. 

## Conclusion

According to the results of this study, treatment with gelfoam impregnated with BTX is an effective and safe method (fewer side effects and pain, compared to injected BTX) in the treatment of patients who have not responded to common therapies for allergic rhinitis.
